# Diabetic Retinal Disease Onset and Progression During Established Semaglutide or Tirzepatide Treatment: A Retrospective Comparative Cohort Study With a Descriptive Regional Nonarteritic Anterior Ischemic Optic Neuropathy (NAION) Review

**DOI:** 10.7759/cureus.118195

**Published:** 2026-10-19

**Authors:** Jamal Muhsen

**Affiliations:** 1 Ophthalmology, Al Dhafra Hospitals, Abu Dhabi, ARE

**Keywords:** diabetic macular edema, diabetic retinopathy, naion, retrospective cohort, semaglutide, tirzepatide

## Abstract

Objectives: This study aims to compare diabetic retinal disease onset or progression between established semaglutide or tirzepatide users and patients without documented exposure to either drug. Secondary exploratory objectives included medication-specific comparisons, adjusted analyses, and visual acuity assessment. Regional nonarteritic anterior ischemic optic neuropathy (NAION) case ascertainment was evaluated separately and descriptively.

Methods: This retrospective cohort reviewed retinal records covering January 1, 2025, through March 31, 2026. Of 342 patients screened, 221 were excluded, leaving 121 records with two bilateral retinal assessments approximately one year apart. Analysis was at the patient-record level. Thirty patients received the same study drug before baseline and throughout follow-up; 91 had no documented exposure to either drug. Incident diabetic retinopathy (DR) and/or diabetic macular edema (DME) was evaluated among records without baseline disease, and progression was evaluated separately among records with baseline disease. Risk ratios (RRs), 95% confidence intervals (CIs), exact tests, and exploratory adjusted models were calculated. A separate regional NAION search covered January 1, 2022, through December 31, 2025, followed by investigator review of identified cases and medication histories.

Results: The combined retinal endpoint occurred in 12/30 exposed and 46/91 unexposed records (40.0% versus 50.5%; RR: 0.79, 95% CI: 0.49-1.28; p = 0.400). Incident disease occurred in 7/14 versus 32/43 records (RR: 0.67; 95% CI: 0.39-1.17), and progression occurred in 5/16 versus 14/48 records (RR: 1.07; 95% CI: 0.46-2.51). Combined event proportions differed across semaglutide, tirzepatide, and unexposed categories (2/14, 10/16, and 46/91; global p = 0.017). No NAION was recorded in the retinal cohort. None of the seven separately identified regional NAION cases had documented study-drug use after investigator review.

Conclusions: No statistically significant association was detected for the combined retinal endpoint, but estimates were imprecise. Medication-specific findings were exploratory and do not establish comparative safety. Regional NAION findings were descriptive and could not quantify or exclude an association.

## Introduction

Type 2 diabetes mellitus is a chronic metabolic disease characterized by impaired insulin action and elevated blood glucose. The World Health Organization estimated that 830 million people were living with diabetes in 2022 and that more than 95% of diabetes cases were type 2 [1]. Obesity is another major metabolic disease; approximately 940 million adults worldwide were living with obesity in 2024. For adults, the World Health Organization defines obesity as a body mass index (BMI) of at least 30 kg/m² [2].

Diabetic retinopathy (DR) and diabetic macular edema (DME) contribute substantially to visual impairment. A systematic review and meta-analysis estimated global prevalence among people with diabetes at 22.27% for DR and 6.17% for vision-threatening DR, corresponding to approximately 103.12 million adults with DR worldwide in 2020 [3]. Nonarteritic anterior ischemic optic neuropathy (NAION) is a separate cause of visual loss. A historical population-based study in Olmsted County, Minnesota, reported an age- and sex-adjusted annual NAION incidence of 10.2 per 100,000; this estimate describes that study population and should not be interpreted as a regional incidence estimate for the United Arab Emirates [4].

Semaglutide and tirzepatide are used to treat type 2 diabetes and obesity. Their ocular outcomes require consideration in populations already at risk of retinal and optic nerve disease. In SUSTAIN-6, semaglutide was associated with more adjudicated retinopathy complications than placebo (hazard ratio: 1.76; 95% confidence interval (CI): 1.11-2.78) [5]. A subsequent analysis suggested that rapid early glycemic improvement contributed to this finding, particularly among patients with pre-existing DR, poor glycemic control, and insulin treatment [6]. Across GLP-1 receptor agonist cardiovascular outcome trials, the overall retinopathy association was not statistically significant, while greater HbA1c reduction was associated with greater retinopathy risk in meta-regression [7].

Observational findings differ by population and retinal endpoint. A tirzepatide cohort reported increased odds of new proliferative DR but lower odds of incident retinopathy among patients without baseline disease [8]. A large GLP-1 receptor agonist cohort found a modest increase in incident DR without a corresponding increase in major progression endpoints among patients with pre-existing DR [9]. A 2026 systematic review emphasized heterogeneity in baseline disease, retinal outcome definitions, ascertainment, and follow-up [10].

NAION has also been associated with semaglutide in observational studies [11,12]. In June 2025, the European Medicines Agency concluded that NAION should be included as a very rare adverse effect of semaglutide medicines [13]. A 2026 meta-analysis reported an increased relative hazard with low certainty because the evidence was observational and subject to bias and confounding [14].

The primary objective of the present study was to compare the combined patient-level endpoint of diabetic retinal disease onset or progression between established semaglutide or tirzepatide users and patients without documented exposure to either drug in routine Al Dhafra care. Medication-specific comparisons, adjusted analyses, and visual acuity assessment were secondary exploratory objectives. Exposure represented treatment established before baseline and continued throughout follow-up; the study did not directly evaluate the period immediately after treatment initiation. A separate descriptive regional review examined recorded NAION diagnoses and the medication histories of identified cases.

## Materials and methods

Ethics approval and informed consent

This study was approved by the Al Dhafra Hospitals Research Ethics Committee (ADHREC; reference: ADH REC 311025) on November 25, 2025. The study involved a retrospective review of existing clinical records without patient contact or research-related interventions. A de-identified dataset was used for analysis.

Study design and setting

This retrospective observational study examined diabetic retinal outcomes in routine clinical care at Al Dhafra Hospitals, Abu Dhabi, United Arab Emirates. The retinal record-review period was January 1, 2025, through March 31, 2026. A separate regional search examined recorded diagnoses of NAION between January 1, 2022, and December 31, 2025.

The principal retinal analysis compared two documented bilateral ophthalmic assessments per patient record. The first assessment defined baseline and the second defined follow-up. According to investigator review, the assessments were approximately one year apart, with variation between patients. The calendar record-review period was distinct from this individual follow-up interval. Exact assessment dates were not retained in the de-identified analytic extract; therefore, the individual follow-up distribution, person-time, and time to retinal change could not be calculated.

The unit of analysis was the patient record. Findings from either eye contributed to a single patient-record-level endpoint; the two eyes were not treated as independent observations. The separate regional NAION cases were summarized descriptively and were not pooled with the paired retinal cohort.

The primary objective was to compare the combined endpoint of incident diabetic retinal disease or progression of baseline disease between records with established, continuing semaglutide or tirzepatide exposure and records without documented exposure to either drug. Component outcomes were analyzed separately. Medication-specific comparisons, adjusted models, and visual acuity analyses were exploratory. The NAION component was descriptive case ascertainment rather than a comparative incidence or medication-safety analysis. The manuscript was organized with reference to the Strengthening the Reporting of Observational Studies in Epidemiology (STROBE) reporting guidance [15].

Eligibility and cohort assembly

The source protocol covered adults with type 2 diabetes mellitus and/or obesity. Obesity was defined as a body mass index of at least 30 kg/m², consistent with the World Health Organization definition [2]. This threshold applied to the obesity eligibility pathway; patients eligible through type 2 diabetes were not required to meet the obesity threshold.

Eligibility for the paired retinal analysis required two documented bilateral ophthalmic assessments and an ascertainable study-drug exposure category. The principal investigator confirmed clinical eligibility by reviewing the source medical records. Of 342 patients screened, 221 were excluded, and 121 records were included in the analytic cohort. A reason-specific breakdown of the 221 exclusions was unavailable for this report.

Protocol exclusions included prior NAION or other optic neuropathy, age-related macular degeneration, retinal vein occlusion, advanced renal impairment, systemic corticosteroid exposure during follow-up, pregnancy, active malignancy, and uncontrolled hypertension. Patients receiving intravitreal injections were not included.

The de-identified analytic extract did not contain age, sex, body mass index, blood pressure, diabetes duration, or an indicator distinguishing eligibility through diabetes from eligibility through obesity. These characteristics could not be tabulated or independently rechecked from the analytic file. In particular, diabetes status within the baseline retinal disease-free stratum could not be independently verified from the extract alone.

All 121 records had values in the 14 source analysis fields. No exact duplicate rows were identified. A stable patient identifier was absent from the de-identified extract; therefore, exclusion of repeated patient records beyond identical field combinations relied on the original clinical record assembly. The cohort was not assumed to represent a complete census or consecutive sample of all eligible regional patients.

Exposure classification

The exposed group comprised patients taking semaglutide or tirzepatide before the baseline retinal assessment and continuing the same agent throughout the interval between the paired assessments. According to the investigator, no switching between the two study drugs occurred during this interval. The finalized exposure classification comprised 14 semaglutide records and 16 tirzepatide records.

The comparison group comprised 91 records without documented exposure to either semaglutide or tirzepatide during the assessment interval. Absence of these two study drugs was not interpreted as absence of all incretin-based or other glucose-lowering therapy.

Exact treatment initiation dates, treatment duration before baseline, dose, adherence, and concomitant glucose-lowering medications were unavailable for analysis. Exposure therefore represented established treatment rather than treatment initiation, and the study did not directly evaluate ocular outcomes immediately after starting either drug.

Retinal outcome definitions and verification

Baseline diabetic retinal disease was defined as documented DR and/or DME in either eye. Absence of baseline disease required both eyes to have neither condition.

The investigator's incident-case definition required no previous DR or DME, followed by newly documented DR, DME, or both in at least one eye at follow-up. The analytic disease-free stratum was identified from bilateral baseline findings. A separate prior-history field was unavailable, so absence of disease before baseline could not be independently verified from the analytic extract. Patients with bilateral disease or both DR and DME contributed only one incident event.

Among patients with baseline retinal disease, progression comprised a higher documented DR category, newly documented DME, or a newly documented diabetic retinal complication, such as vitreous hemorrhage, in either eye. The recorded clinical DR categories were mild, moderate, and severe nonproliferative DR and proliferative DR. The extract could not quantify worsening of pre-existing DME within an unchanged category.

The primary combined endpoint joined incident disease in the baseline disease-free stratum with progression in the baseline disease stratum. Because these components represent different clinical processes, they were also reported separately. Outcomes were based on clinical documentation rather than masked central grading of standardized retinal images. No retrospective application of a standardized image-grading protocol was claimed.

During data verification, five positive progression flags with unchanged bilateral retinal categories were identified by the principal investigator as recording errors and corrected to no progression. Source fields were preserved separately, and all final analyses used the corrected endpoint. Appendix A documents these corrections and the analysis rules.

Visual acuity assessment

Visual acuity was an exploratory secondary outcome. Quantitative analysis required Snellen measurements in both eyes at both assessments. Snellen values were converted to logMAR using log10(denominator/numerator). Worsening was defined as an increase of at least 0.2 logMAR in either eye between baseline and follow-up.

Seven records with counting-fingers or no-light-perception notation at any assessment were excluded from this secondary comparison: three exposed and four unexposed records. These records remained in the principal retinal cohort. Qualitative visual acuity entries were not assigned arbitrary numerical values.

The quantitative visual acuity analysis therefore included 114 records. Refraction, cataract, and other potential causes of visual acuity change were not controlled, and worsening was not attributed specifically to diabetic retinal disease or study-drug exposure.

NAION ascertainment

Within the paired retinal cohort, NAION ascertainment was based on the recorded clinical diagnosis field. For the separate regional search, electronic records were searched for ischemic optic neuropathy diagnoses during January 1, 2022, through December 31, 2025, using the following ICD-10-CM codes [16] (H47.011: ischemic optic neuropathy, right eye; H47.012: Ischemic optic neuropathy, left eye; H47.013: ischemic optic neuropathy, bilateral; and H47.019: ischemic optic neuropathy, unspecified eye). These codes identify ischemic optic neuropathy and do not independently distinguish arteritic from nonarteritic disease. NAION case classification therefore relied on the documented clinical diagnosis and investigator review of the source records.

The regional search identified seven patients with recorded NAION diagnoses. The principal investigator personally reviewed the clinical records and medication histories of all seven patients and found no documented semaglutide or tirzepatide use in any of them.

This was diagnosis-based case ascertainment. Independent neuro-ophthalmic adjudication was not performed. Completeness depended on diagnostic coding and documentation within the electronic records searched; uncoded diagnoses, care received outside the reviewed system, and incomplete medication histories could affect case ascertainment or exposure classification.

Regional exposed and unexposed population denominators and person-time were unavailable. Consequently, these cases were reported descriptively and were not used to calculate regional incidence, comparative risk, or a causal association with either study drug.

Statistical analysis

Continuous variables were summarized using means and standard deviations for descriptive purposes. No between-group t tests or analysis of variance were performed. Formal normality and variance-homogeneity tests were not performed; the principal comparisons concerned binary outcomes and used exact tests rather than methods requiring normally distributed continuous outcomes or equal group variances.

Binary outcomes were reported as event counts and observed proportions. Unadjusted risk ratios (RRs) compared exposed with unexposed records, with 95% CIs calculated on the logarithmic scale. Two-sided Fisher exact tests were used for the two-group comparisons.

The corrected combined retinal endpoint was the principal comparison. Incident disease and progression were analyzed separately within their respective baseline strata. Medication-category heterogeneity across semaglutide, tirzepatide, and unexposed records was assessed using a two-sided Fisher-Freeman-Halton exact test with fixed margins. This global test did not establish a confirmatory pairwise difference between the two study drugs.

Exact Clopper-Pearson 95% CIs were calculated for the zero-event NAION proportions in the paired retinal cohort. These intervals described observed proportions rather than annualized incidence. A comparative NAION RR was not estimable because both exposure groups had zero events.

Exploratory logistic regression models were fitted separately within the baseline retinal disease-free and baseline disease strata. Each model included binary study-drug exposure, continuous baseline HbA1c, and recorded estimated glomerular filtration rate (eGFR), without interaction terms. Maximum likelihood estimates and Wald 95% CIs were used. Adjusted odds ratios were not interpreted as RRs.

The models provided limited adjustment because the available extract did not capture important demographic, clinical, and treatment-related confounders. Small strata and established treatment use further restricted causal interpretation.

No imputation, propensity-score adjustment, survival analysis, or post hoc power calculation was performed. Study size was determined by the available eligible paired records. A two-sided p-value below 0.05 was used as the statistical significance threshold. No multiplicity adjustment was applied, and secondary analyses were considered exploratory. Calculations used Python, NumPy, and SciPy. Logistic regression coefficients were checked using a second optimization method.

## Results

Cohort assembly and characteristics

Of 342 patients screened, 221 were excluded, and 121 records were included in the paired retinal analysis. Thirty records were exposed to a study drug: 14 to semaglutide and 16 to tirzepatide. Ninety-one records had no documented exposure to either drug.

At baseline, 57 records were free of both DR and DME, comprising 14 exposed and 43 unexposed records. The remaining 64 records had baseline retinal disease, comprising 16 exposed and 48 unexposed records.

All 121 records had values in the 14 source analysis fields. Available baseline characteristics are presented in Table 1. Patient groups by exposure status, baseline retinal disease, and retinal course at follow-up are summarized in Table 2. No exact duplicate rows were found. A stable patient identifier was absent from the de-identified extract, so exclusion of duplicates beyond identical field combinations relied on the original clinical record assembly.

**Table 1:** Available baseline characteristics eGFR: estimated glomerular filtration rate; DR: diabetic retinopathy; DME: diabetic macular edema Values are n (%) or mean ± standard deviation. HbA1c fractions in the source file were converted to percentages. eGFR units are reproduced as recorded; body-surface-area normalization was not specified in the extract. Age, sex, diabetes duration, body mass index, and blood pressure were unavailable for baseline comparison

Characteristic	Exposed (n = 30)	Unexposed (n = 91)
Baseline DR and/or DME	16 (53.3%)	48 (52.7%)
No baseline DR or DME	14 (46.7%)	43 (47.3%)
HbA1c (%)	8.33 ± 1.61	8.88 ± 1.81
eGFR (mL/min as recorded)	94.47 ± 16.15	96.95 ± 18.42
Serum creatinine (µmol/L)	63.83 ± 14.33	78.65 ± 15.66
Semaglutide	14 (46.7%)	0
Tirzepatide	16 (53.3%)	0

**Table 2:** Patient groups by exposure and retinal course DR: diabetic retinopathy; DME: diabetic macular edema Baseline disease means DR and/or DME. Groups are mutually exclusive and sum to 121. No recorded progression does not necessarily mean unchanged visual acuity or the absence of other ocular disease

Exposure	Baseline disease	Course at follow-up	n
Exposed	Absent	Remained absent	7
Exposed	Absent	Incident disease	7
Exposed	Present	No progression	11
Exposed	Present	Progression	5
Unexposed	Absent	Remained absent	11
Unexposed	Absent	Incident disease	32
Unexposed	Present	No progression	34
Unexposed	Present	Progression	14

Primary retinal outcome

The corrected combined retinal endpoint occurred in 12/30 exposed and 46/91 unexposed records (40.0% versus 50.5%; RR: 0.79, 95% CI: 0.49-1.28; p = 0.400) (Table 3). The observed absolute difference was -10.5 percentage points. The CI included both a decrease and an increase in risk.

**Table 3:** Principal retinal outcomes DR: diabetic retinopathy; DME: diabetic macular edema; RR: risk ratio for exposed versus unexposed; CI: confidence interval Incident and progression denominators are the baseline disease-free and baseline-disease strata, respectively. All CIs include 1.0. The combined CI remains compatible with a clinically meaningful decrease or increase in risk

Outcome	Exposed	Unexposed	RR (95% CI)	Fisher p
Any onset or progression	12/30 (40.0%)	46/91 (50.5%)	0.79 (0.49 to 1.28)	0.400
Incident DR and/or DME	7/14 (50.0%)	32/43 (74.4%)	0.67 (0.39 to 1.17)	0.107
Progression of baseline disease	5/16 (31.3%)	14/48 (29.2%)	1.07 (0.46 to 2.51)	1.000

Component retinal outcomes

Among records without baseline DR or DME, incident disease occurred in 7/14 exposed and 32/43 unexposed records (50.0% versus 74.4%; RR: 0.67; 95% CI: 0.39-1.17; p = 0.107).

Among records with baseline retinal disease, progression occurred in 5/16 exposed and 14/48 unexposed records (31.3% versus 29.2%; RR: 1.07; 95% CI: 0.46-2.51; p = 1.000) (Table 3). These were observed proportions between paired assessments rather than population incidence rates.

Exploratory medication-specific, adjusted, and visual acuity analyses

Combined endpoint proportions were 2/14 (14.3%) for semaglutide, 10/16 (62.5%) for tirzepatide, and 46/91 (50.5%) for unexposed records (global exact p = 0.017) (Table 4). These small, nonrandom groups were not balanced or adjusted for agent-specific confounders. The global test described heterogeneity across all three categories and did not establish a causal or confirmatory pairwise difference between semaglutide and tirzepatide. In exploratory adjusted models, the exposure association was an adjusted odds ratio of 0.33 for incident disease (95% CI: 0.09-1.20; p = 0.093) and 1.02 for progression (95% CI: 0.29-3.62; p = 0.970) (Table 5).

**Table 4:** Exploratory outcomes by medication category Incident disease and progression use different baseline strata. No switching occurred between the paired visits according to the investigator. Medication-specific results are exploratory and cannot establish an intrinsic difference in ocular safety

Category	Records	Any event	Incident disease	Progression
Semaglutide	14	2/14 (14.3%)	1/6 (16.7%)	1/8 (12.5%)
Tirzepatide	16	10/16 (62.5%)	6/8 (75.0%)	4/8 (50.0%)
Unexposed	91	46/91 (50.5%)	32/43 (74.4%)	14/48 (29.2%)

**Table 5:** Exploratory adjusted and visual acuity analyses aOR: adjusted odds ratio from models including exposure, baseline HbA1c, and eGFR; DR: diabetic retinopathy; DME: diabetic macular edema Model p-values are two-sided Wald tests; the visual-acuity p-value is Fisher exact. Acuity worsening was at least 0.2 logMAR in either eye. Acuity change was not attributed specifically to DR or to drug exposure

Analysis	Records	Exposure estimate (95% CI)	p
Adjusted incident DR/DME	57; 39 events	aOR: 0.33 (0.09 to 1.20)	0.093
Adjusted progression	64; 19 events	aOR: 1.02 (0.29 to 3.62)	0.970
Snellen-only acuity worsening	114; 15 events	RR: 2.15 (0.84 to 5.49)	0.188

The quantitative visual acuity analysis included 114 records. Worsening occurred in 6/27 exposed and 9/87 unexposed records (22.2% versus 10.3%; RR: 2.15; 95% CI: 0.84-5.49; p = 0.188) (Table 5). Three exposed and four unexposed records were excluded from this secondary analysis because of qualitative low-vision notation. Visual acuity change was not attributed specifically to DR or drug exposure.

NAION findings

No NAION was recorded among the 121 analytic retinal records, including the 30 exposed records. The two-sided exact upper 95% CI limit for the exposed group was 11.6%, illustrating the imprecision of a zero count in a small sample. A comparative NAION RR was not estimable because both exposure groups had zero events (Table 6).

**Table 6:** Recorded NAION in the paired retinal cohort CI: confidence interval; NAION: nonarteritic anterior ischemic optic neuropathy Clopper-Pearson CIs apply to observed proportions in the paired cohort. They are not annualized rates and do not describe the separate regional case search

Group	Events/records	Observed proportion	Exact 95% CI
All records	0/121	0%	0% to 3.0%
Exposed	0/30	0%	0% to 11.6%
Unexposed	0/91	0%	0% to 4.0%

The separate regional search identified seven patients with recorded NAION diagnoses between January 1, 2022, and December 31, 2025. Following personal review of all seven clinical records and medication histories, the principal investigator found no documented semaglutide or tirzepatide use in any of these patients. Because regional exposure denominators and person-time were unavailable, these findings supported descriptive statements about identified cases rather than regional incidence or comparative risk estimates.

## Discussion

Principal findings

In this selected retrospective cohort, the combined endpoint of incident diabetic retinal disease or progression of baseline disease occurred in 12/30 exposed records and 46/91 records without documented study-drug exposure (40.0% versus 50.5%; RR: 0.79, 95% CI: 0.49-1.28; p = 0.400). No statistically significant association was detected in the principal comparison, but the confidence interval remained compatible with a clinically meaningful decrease or increase in risk. Separate analyses of incident disease and progression were also inconclusive.

The global comparison across semaglutide, tirzepatide, and unexposed records showed heterogeneity in observed endpoint proportions. This exploratory finding did not establish a causal or confirmatory pairwise difference between the two study drugs. The NAION findings were descriptive and did not provide a comparative incidence or medication-safety analysis.

Strengths

Strengths included paired bilateral retinal assessments, analysis at the patient-record level rather than treating the two eyes as independent observations, separate reporting of incident disease and progression, transparent documentation of investigator-confirmed endpoint corrections, and presentation of effect estimates with confidence intervals. Personal review of the clinical records and medication histories of the seven regional NAION cases provided additional descriptive information about documented study-drug exposure.

Interpretation of the retinal findings

The findings concern patients already receiving semaglutide or tirzepatide when retinal follow-up began. They, therefore, cannot resolve early retinal worsening immediately after treatment initiation or following rapid improvement in glycemic control. Treatment initiation dates and serial HbA1c measurements were unavailable. The post hoc SUSTAIN-6 analysis and the cardiovascular outcome trial meta-regression linked retinal outcomes to glycemic change in susceptible patients [6,7]. This potential mechanism could not be evaluated using a single baseline HbA1c measurement.

The combined endpoint also differed from the sight-threatening retinopathy complications assessed in major trials. Buckley et al. reported increased odds of incident proliferative DR with tirzepatide while observing a different association among patients initially free of retinopathy [8]. Ramsey et al. reported a modest association with incident DR without a corresponding increase in major progression outcomes among patients with pre-existing DR [9]. These estimates should not be compared directly with the high observed incident-disease proportion in the present selected ophthalmic cohort. Differences in sampling, baseline disease, assessment methods, and endpoint definitions can produce substantially different absolute proportions. A systematic review similarly emphasized heterogeneity in the assessment and reporting of retinal outcomes [10].

Additional studies illustrate the importance of comparator selection and study design. Kapoor et al. reported differing retinal associations according to the comparator treatment in their meta-analysis [17]. The OHDSI network study by Cai et al. found no increased risk of proliferative DR or treatment-requiring DR/DME with semaglutide compared with the other medications evaluated [18]. Shah et al. reported lower risks of several retinal outcomes and related interventions among tirzepatide users compared with lifestyle-intervention controls [19]. These observational findings provide context but do not establish comparative safety in the present cohort. Differences in baseline retinal status, population characteristics, treatment selection, and outcome definitions limit direct comparisons between studies.

The observed combined endpoint proportions were 2/14 for semaglutide, 10/16 for tirzepatide, and 46/91 for unexposed records. Although the global exact test was statistically significant, the groups were small and nonrandomized, and the analysis did not adequately characterize or control agent-specific confounding. Unmeasured diabetes duration, treatment selection, concomitant medications, and glycemic trajectories could contribute to the numerical differences. Secondary analyses were conducted without multiplicity adjustment. A larger study with consistent retinal grading and an appropriate comparator is required before drawing an agent-specific conclusion.

Adjustment for baseline HbA1c and recorded eGFR did not identify a statistically significant exposure association in either baseline retinal stratum. These models provided limited adjustment and did not establish absence of an effect. Similar observed progression proportions likewise do not demonstrate equivalence, and neither the principal analysis nor the exploratory models established medication safety.

Outcome ascertainment and visual acuity

The five progression-flag corrections were confirmed by the principal investigator and documented in the Appendices. Preservation of the original fields and use of the corrected endpoint improved transparency, but this verification did not replace masked clinical adjudication. Retinal outcomes remained dependent on the available clinical documentation. In particular, the extract could not quantify worsening of existing DME within an unchanged category. Future studies should distinguish changes in DR grade, incident DME, worsening DME, and sight-threatening retinal complications.

The exploratory visual acuity analysis was imprecise and vulnerable to confounding by other ocular conditions. Restricting the quantitative comparison to records with bilateral Snellen measurements at both assessments avoided arbitrary numerical assignments to counting-fingers and no-light-perception entries, but excluded seven records from this secondary analysis. The observed visual acuity difference could not be attributed specifically to diabetic retinal disease or study-drug exposure because refraction, cataract, and other causes of acuity change were not controlled.

Interpretation of the NAION findings

No NAION was recorded in the paired retinal cohort. The separate regional search identified seven patients with recorded NAION diagnoses between January 1, 2022, and December 31, 2025. Personal review of their clinical records and medication histories found no documented semaglutide or tirzepatide use. These observations do not quantify or exclude an association with either drug because regional exposure denominators and person-time were unavailable.

Published evidence should be interpreted separately from this descriptive case search. Hathaway et al. reported an association between semaglutide exposure and NAION in a single-center matched cohort [11]. Cai et al. subsequently evaluated the association across multiple databases and reported a smaller relative increase in NAION incidence [12]. These differing estimates illustrate the influence of ascertainment, population, and study design. A 2026 meta-analysis also reported an association while emphasizing the uncertainty of the observational evidence [14]. The present descriptive findings do not overturn that evidence.

Clinical interpretation should preserve the distinction between the two study drugs. The European Medicines Agency classified NAION as a very rare adverse effect of semaglutide and advised discontinuation of semaglutide if NAION is confirmed [13]. That guidance concerns semaglutide and should not be extrapolated to tirzepatide based on the present dataset. This study provides no basis for a class-wide causal conclusion.

Limitations and generalizability

The principal limitations were the small exposed cohort, observational treatment assignment, incomplete demographic and treatment covariates, and absence of an active-comparator design. These limitations restricted confounding adjustment, causal interpretation, and generalizability.

The protocol permitted eligibility through type 2 diabetes and/or obesity, but the analytic extract did not encode the eligibility pathway. Diabetes status within the incident-disease stratum therefore could not be independently verified or used to restrict that stratum from the extract alone. Clinical eligibility relied on investigator review of the source records. This limitation affects interpretation of the incident diabetic retinal disease endpoint.

Although the screening and exclusion totals were established as 342 and 221, respectively, a reason-specific exclusion breakdown was unavailable for this report. Requiring paired ophthalmic assessments may have selected patients receiving closer eye care. Excluding patients receiving intravitreal injections may have underrepresented more severe or treated retinal disease. The cohort should therefore not be interpreted as representative of all eligible regional patients.

Exposure represented established treatment before baseline rather than treatment initiation. Requiring continued use of the same drug may have omitted patients who discontinued treatment after an early adverse event. Baseline retinal findings among established users may already have reflected changes occurring before the first assessment. The combined endpoint included incident disease and progression, which are clinically distinct. Component analyses were reported separately, but their small denominators limited precision.

The retinal record-review period was January 1, 2025, through March 31, 2026. This calendar period was distinct from the approximately one-year interval between paired assessments. Exact individual assessment and treatment initiation dates were unavailable in the analytic extract, preventing calculation of the follow-up distribution, person-time, or time to retinal change.

Outcome assessment was not masked, and central standardized review of retinal images was not performed. Exact-row duplicate checking could not exclude all repeated patient records in the absence of stable patient identifiers. Complete values in the analyzed fields did not imply complete clinical information.

The regional NAION search used specified ischemic optic neuropathy codes followed by investigator review of documented diagnoses and medication histories. Completeness nevertheless depended on coding and documentation within the electronic records searched. Uncoded diagnoses, care received outside the reviewed system, and incomplete medication histories could have affected case ascertainment or exposure classification. Independent neuro-ophthalmic adjudication was not performed.

Regional exposure denominators and person-time were unavailable. The absence of documented study-drug use among the seven identified NAION cases therefore cannot quantify or exclude an association with either drug. Larger longitudinal studies should retain coded patient identifiers, precise assessment and treatment dates, validated clinical diagnoses, retinal images, injection histories, and longitudinal systemic treatment data.

## Conclusions

In this 121-record cohort, established semaglutide or tirzepatide exposure was not significantly associated with the combined diabetic retinal endpoint over approximately one year (RR: 0.79; 95% CI: 0.49-1.28). Imprecise estimates and residual confounding prevent conclusions of equivalence or safety; medication-specific differences remain exploratory. No NAION was recorded in the retinal cohort, and none of the seven separately identified regional cases had documented study-drug use, but these descriptive findings cannot quantify or exclude a NAION association.

## Appendices

Reporting gaps relevant to STROBE [15] include exact individual encounter dates and the follow-up distribution; treatment initiation dates and detailed exposure histories; reason-specific exclusion counts; demographic characteristics and the diabetes-versus-obesity eligibility indicator; complete confounder histories; and independent outcome adjudication. Screening and exclusion totals are reported as 342 and 221, respectively. The retinal review calendar window and the regional NAION search period and diagnostic codes are specified in Methods. Remaining gaps are acknowledged explicitly rather than replaced by estimated or inferred patient data.

**Table 7:** Investigator confirmed corrections from progression to no progression DR: diabetic retinopathy; DME: diabetic macular edema; NPDR: nonproliferative DR; PDR: proliferative DR Source row numbers are sequential, anonymous analytic record numbers, not patient identifiers. The final endpoint is No for all five records

Source row	Exposure	Right eye at both visits	Left eye at both visits
39	Unexposed	PDR + DME	PDR + DME
40	Unexposed	PDR + DME	PDR + DME
55	Tirzepatide	Moderate NPDR + DME	Moderate NPDR + DME
90	Unexposed	PDR + DME	PDR + DME
117	Unexposed	Mild NPDR + DME	No DR or DME
